# Feasibility and Acceptability of Antenatal Hepatitis C Screening: A Pilot Study

**DOI:** 10.1155/2024/7696410

**Published:** 2024-08-27

**Authors:** Joseph Valamparampil, Jaswant Sira, Maxine Brown, Saket Singhal, Deirdre Kelly

**Affiliations:** ^1^ Liver Unit Birmingham Women's and Children's Hospital NHS Foundation Trust, Birmingham, UK; ^2^ Sandwell & West Birmingham Hospitals NHS Trust, West Bromwich, UK; ^3^ University of Birmingham, Birmingham, UK

## Abstract

**Introduction:**

Hepatitis C virus (HCV) is not currently included in the United Kingdom routine antenatal screening program, but the latest guidelines from the Centers for Disease Control and Prevention, American Association for the Study of Liver Diseases, and Infectious Diseases Society of America recommend HCV screening for all pregnant women during each pregnancy. The aim of this study was to collect qualitative data on the feasibility and acceptability of antenatal HCV screening in pregnant women at the time of routine antenatal screening at 12 weeks, to estimate patient knowledge about HCV and identify the prevalence of HCV infection in antenatal women.

**Methods:**

This was a pilot study targeting a single hospital-based antenatal clinic in Birmingham, initially conducted for eight weeks with a further extension of the study period to enhance recruitment to meet the feasibility target of 500 patients. Data collected included demographic and epidemiological details. Pregnant women attending the antenatal unit were given information regarding HCV and antenatal screening for HCV prior to their initial antenatal visit. During the antenatal visit, research nurses provided further information about the study and HCV infection. Consent was obtained for taking part in the study and testing for HCV using blood samples taken at the same time as other routine antenatal screening blood tests. All women who agreed to participate in the study were asked to complete an acceptability and knowledge questionnaire. All women had HCV antibody testing as the primary screening assay. The test result was communicated in writing to the women and their general practitioner. Confirmatory positive antibody tests were followed up with quantitative HCV PCR and genotype analysis. The outcomes of testing were no evidence of HCV infection and evidence of past HCV infection or current HCV infection.

**Results:**

Five hundred and forty-nine women were approached in the antenatal clinic; 30 women refused consent while 29 women were excluded from the study (blood tests not performed after consenting, age less than 18 years, and consent form lost). Four hundred and ninety women were included in the study. The median age of the study population was 29 years (range, 18–46). Knowledge about blood-borne viruses was limited; 75% of women had some understanding about antenatal hepatitis B (HBV) and human immunodeficiency virus (HIV) testing. Previous awareness about hepatitis C was reported by 55%. Ninety-one percent of women found the information they were given about hepatitis C helpful. Ninety-six percent of the women included in this study found the counselling they received about HCV useful and felt that the delivery of this information was carried out in an acceptable manner. Once given information about HCV, 99% felt that universal screening for HCV should be implemented. HCV antibody was negative in 489 women. One patient with a positive HCV antibody (prevalence: 0.2%) had a negative HCV PCR.

**Conclusion:**

Routine antenatal screening for HCV is not currently recommended in the UK. Our study suggests that antenatal HCV screening would be both feasible and acceptable to most pregnant women attending antenatal clinics. Though the awareness of HCV was low, with appropriate counselling and communication, 99% of pregnant women were in favor of antenatal screening for HCV. Antenatal screening would identify HCV-positive mothers and allow follow-up of their infants so that any infected mothers and infants could be offered effective curative therapy and prevent the progression of liver disease. The inclusion of HCV antenatal screening would complete the blood-borne virus profile and enhance the WHO target to eliminate HCV in the UK.

## 1. Introduction

The World Health Organization (WHO) estimates that 1% of the global population lives with chronic hepatitis C (HCV) infection [1]. HCV is an important cause of chronic liver disease worldwide [1]. In 2018, there were an estimated 3.26 million children and adolescents (≤18 years) living with chronic HCV [2]. WHO Global Health Sector Strategy on viral hepatitis aims to eliminate HCV as a public health threat by 2030 [1]. One of the challenges in achieving this goal is that globally, only 20% of infected individuals are aware of their HCV status [1]. The United Kingdom (UK) has a low prevalence of HCV infection. The prevalence of chronic HCV infection in the UK among adults aged ≥16 years was 0.17% in 2021; this equates to approximately 92,900 (76,000–116,800) individuals [3]. HCV prevalence has declined by 47% as compared to 2015 [3]. Further work is required to attain the target set by WHO for 2030 of at least 80% of people with chronic HCV to be diagnosed and accessing treatment [3, 4].

The risk of mother-to-child transmission of HCV from infected mothers is 5–8% [5]. Vertical transmission accounts for only 0.4% of new infections; however, this is currently the main mode of transmission in children. HCV infection in children is usually asymptomatic but can present in later life with complications of chronic liver disease and is associated with increased lifetime risk of cirrhosis and liver cancer [6]. The updated 2022 WHO guideline has recommended treatment using pan genotypic direct-acting antiviral (DAA) regimens in all children aged ≥3 years with chronic HCV infection, regardless of stage of disease [2]. This is complementary to genotype-specific DAA therapy.

The aim of this study was to collect qualitative data on the feasibility and acceptability of antenatal HCV screening in pregnant women in a single antenatal clinic in Birmingham, to estimate women's knowledge about HCV and the acceptability and feasibility of screening, and to identify the prevalence of HCV infection in a representative sample of pregnant women.

## 2. Subjects and Methods

This was a pilot study targeting a single hospital-based antenatal clinic with approximately 100 antenatal screening visits per week in Birmingham. As this was a pilot study, screening 500 women was considered sufficient for the feasibility and acceptability of antenatal HCV screening, to evaluate women's knowledge about HCV and estimate the prevalence rate. The study was initially conducted for eight weeks, and a further extension was granted for one year to enhance recruitment to meet the feasibility target of 500 patients. The conduct of the study was in accordance with the principles and conditions of Good Clinical Practice and the World Medical Association Declaration of Helsinki. The study was approved by National Health Service Research Ethics Committee. Written informed consent to participate in the study was obtained from all participants and all patient identifiable data were anonymised. Study data were collated and maintained in accordance with national guidelines. A comprehensive database was constructed to collect and store data in accordance with research governance recommendations. Women who declined or were unable to provide informed consent, including those who were <18 years of age, were excluded from the study.

Study information for pregnant women was posted along with their booking appointment prior to their initial antenatal visit. This included written information regarding blood-borne viruses (BBVs), antenatal screening for HCV, and testing and prevention of infection. During the antenatal visit, research nurses (one nurse had Asian bilingual skills) discussed further information about the study and obtained consent. All women who agreed to participate in the study were requested to complete an acceptability questionnaire (designed by the research team) which explored their understanding of antenatal HCV screening, opinion on the acceptability of screening, and views on the counselling they had received. The questions were divided into two sets; the first set evaluated the knowledge of BBV and testing with specific focus on HCV. The second set assessed the acceptability of the information given (Table 1). The questionnaire was not translated into other languages, but when required, interpreters were used. Initially, patients took the questionnaire away from clinic and returned it to the research nurses by post. Unfortunately, several questionnaires were not returned and so after the first 70 patients, to increase data capture, the study design was changed so that the study was discussed face to face, information leaflets were provided, and questionnaires were completed in the antenatal clinic.

Testing for HCV was from blood samples taken at the same time as other routine screening blood tests. Abbott Architect anti-HCV assay which is a chemiluminescent microparticle immunoassay for the qualitative detection of antibody in serum and plasma was used as the primary screening assay at the same time as antenatal blood tests. Confirmatory testing for HCV antibodies was done using VIDAS anti-HCV platform (bioMérieux). Confirmatory positive antibody tests were to be followed up with quantitative HCV PCR and genotype analysis. The outcomes of testing were as follows:

1. Anti-HCV not detected and reported as “Anti-HCV not detected, no evidence of HCV infection.”
2. Anti-HCV detected, HCV RNA not detected and reported as “Anti-HCV detected, HCV RNA not detected, compatible with past HCV infection.”
3. Anti-HCV and HCV RNA detected and reported as “Anti-HCV and HCV RNA detected, compatible with current HCV infection.”

HCV-RNA-positive women were to be referred to specialist liver services for further management. All women with blood tests negative for HCV were informed by letter and offered the opportunity to discuss the result with a member of the research team by telephone or a face-to-face appointment. Study participants with a blood test positive for past or current HCV infection were informed of their status by the research team by telephone. An appointment was offered for repeat blood tests to confirm infection, with appropriate counselling and follow-up at a specialist hepatitis clinic. Family counselling and referral of the newborn children to a specialist paediatric unit was planned to be offered. Information, support, and counselling were provided by trained medical professionals experienced in the management of patients with HCV. The obstetric team managing the delivery of HCV-infected mothers and their general practitioner were also informed. All blood test results and outcomes were communicated to the general practitioner.

## 3. Results

**Figure 1:**
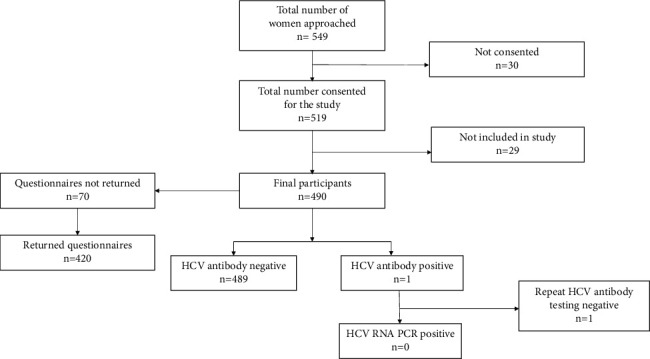
Flow diagram of patients approached to take part in antenatal screening for hepatitis C and outcomes.

Five hundred and forty-nine women were approached in the antenatal clinic to give information about the study. 30 women refused consent. The reasons were as follows: did not want additional blood tests (*n* = 3), language barrier (*n* = 14), and refused consent (*n* = 13). Of the 519 women who consented, 29 women were not included in the study. The reasons were blood tests not performed after consenting (*n* = 27), age <18 years (*n* = 1), and consent form lost (*n* = 1). The final number of participants included in the study who completed both questionnaires and HCV testing as per study protocol was 490. Since 70 questionnaires were not returned in the initial part of the study, information on the acceptability data is available only in 420 participants (Figure 1).

The median age of the study population was 29 years (range, 18–46 years). The main ethnicity groups included Caucasian (40%), Asian (40%), African (10%), and Afro-Caribbean (10%). Out of the 490 patients who participated in the study, 489 were HCV antibody negative. One patient with a positive HCV antibody (prevalence: 0.2%) had a negative HCV RNA (Figure 1).

In general, knowledge about BBV was limited with 75% of women having some understanding about antenatal hepatitis B virus (HBV) and human immunodeficiency virus (HIV) (Table 1). Only 55% of our population sample were previously aware of HCV. Ninety-one percent of women found the information they were given about HCV helpful. Ninety-six percent of the women included in this study found the counselling they received about HCV useful and felt that the delivery of this information was carried out in an acceptable manner. Once given information about HCV, 99% felt that universal screening for HCV should be implemented.

## 4. Discussion

This pilot study showed that routine antenatal HCV screening was feasible and acceptable to the target population in the UK once the information was appropriately discussed. Our study met its feasibility target for recruitment of 500 patients in a one-year period and the process of the study was acceptable to most of the women. The majority had heard about HIV and HBV, and only 55% of our study participants had previous knowledge of HCV. However, following counselling and discussion, 99% were in favor of universal antenatal screening for HCV.

The study participants' response to the questionnaire was largely positive and there was <2% refusal rate for antenatal HCV testing. The seroprevalence of HCV antibodies was 0.2% (Figure 1). The HCV antibody prevalence reported in our study was similar to previous studies in the UK (<1%) [7–10]. The incidence might be higher in women residing in socioeconomically deprived areas of UK and areas with high prevalence of injectable drug users [8, 10].

Seventy-five percent of people living with chronic HCV remain unaware of their infectious status [3]. The UK has already surpassed the WHO absolute target of mortality ≤2 per 100,000 and exceeded the 2020 WHO target of 10% reduction in mortality compared to 2015 [3]. Additional decrease in the mortality will need identifying and treating those living with chronic HCV infection. Also, early diagnosis of HCV infection is important for the most effective follow-up care and treatment. Current UK guidelines for the detection of HCV involve testing high-risk individuals including injectable drug users. Access to sterile injecting equipment and opioid agonist therapy are important harm reduction interventions being facilitated [3].

Currently, antenatal screening for HCV is risk based, unlike routine screening for HBV and HIV. It is unlikely to identify all the HCV-infected mothers as 40–70% of HCV-infected pregnant women may not report major risk factors [11, 12]. The rationale for not including antenatal screening for HCV in pregnant women in the UK is because there is currently no therapy to prevent transmission, as there are no approved antiviral treatments for infected pregnant women and there are no effective vaccines against HCV. Prevalence of HCV infection in pregnant women in the UK is uncertain and further large studies are needed [11].

Prior to the era of DAAs, antenatal screening of asymptomatic pregnant women for HCV was not considered cost-effective [13]. A modelling study of cost-effectiveness of treatment with DAA in pregnancy after universal prenatal HCV screening demonstrated that it was cost-effective and HCV-infected pregnant women lived 1.21 years longer and had 16% lower HCV attributable mortality and increased identification of infants exposed to HCV at birth from 44% to 92% [14]. HCV in pregnancy has also been associated with adverse outcomes such as higher risk of pregnancy-induced hypertension, antepartum haemorrhage, preeclampsia, gestational diabetes, and miscarriage [11].

Antenatal screening can be a powerful tool in the fight towards elimination of HCV as a public health threat by 2030. DAAs are approved for use in children ≥3 years of age and there are multiple ongoing completed clinical trials evaluating the safety and efficacy of DAAs in pregnancy and the peripartum period [15, 16]. The first published clinical trial of HCV treatment during pregnancy with ledipasvir-sofosbuvir showed that the combination was safe and effective without clinically meaningful differences in drug exposure among pregnant versus nonpregnant women with 100% cure at 12 weeks after completion of treatment and absent mother-to-child transmission at 12 months follow-up [15]. In a study analyzing the preferences of pregnant women with HCV regarding potential DAA treatment during pregnancy, 60% reported that they would be willing to take antepartum DAA therapy if it lowered the risk of perinatal transmission [17]. Yee et al. analyzed the barriers to the inclusion of pregnant women in HCV research and treatment programs in the United States; the women's perceived barriers included cost and access, insufficient provider knowledge and communication challenges, safety concerns, psychological barriers, and unclear rationale for treatment during pregnancy [18]. However, women were well informed about HCV and largely desirous of treatment [18].

Ledipasvir-sofosbuvir combination was well tolerated and highly effective in children 6–12 years old with chronic HCV with sustained virologic response 12 weeks after therapy of 99% [16]. Since the latest WHO recommendation is to treat all children aged ≥3 years with chronic HCV infection using pan genotypic DAA regimens, universal antenatal screening for HCV during pregnancy will enable early identification and treatment of children and mothers.

Our study has shown that antenatal HCV screening is feasible and acceptable to expectant mothers in the UK. The group who refused to participate in the study were mainly of Asian-Pakistani or Eastern European and African origin. The women who refused consent were recent arrivals to the UK and had significant language barriers. The Asian women who did not understand English had no knowledge of BBV. Many had seen relatives with jaundice; however, they were not aware of HBV and HCV. Additionally, they refused to participate without their husband's or in-laws' agreement. Women from Europe and Africa who participated in the study who refused testing knew about HIV and HBV but not about HCV. Some stated they already had the test done but did not want to disclose the result to the research team. The risk of HCV infection in pregnant women of those who refuse may be missed as identified in previous BBV studies [19].

The Centers for Disease Control and Prevention (2020), American Association for the Study of Liver Diseases (2022), and Infectious Diseases Society of America (2022) guidelines have recommended HCV screening for all pregnant women during each pregnancy (ideally at initial visit), except in settings where the prevalence of HCV infection is <0.1% [20]. In contrast, the UK National Screening Committee recommended in 2018 that screening for HCV in pregnancy does not meet the criteria for a systematic population screening program due to the lack of interventions to prevent vertical transmission and the lack of benefit with respect to the management of maternal disease during pregnancy [11].

The report also highlighted a lack of knowledge regarding the seroprevalence of HCV in the contemporary pregnant population in the UK. Our study has sought to address this as well as evaluating acceptability and feasibility. We identified that only 55% of women knew about HCV and their knowledge of BBV was limited, indicating the need for a more focused approach when screening for these viruses.

A potential limitation of this study is that patients were recruited from a single hospital antenatal clinic, and thus the study population may not be representative of the general antenatal population, resulting in an inaccurate estimated prevalence of HCV infection. Another limitation was the initial significant number of unreturned questionnaires (14%).

In conclusion, the prevalence of HCV antibodies in our study population of pregnant women was 0.2%. Our study has demonstrated that antenatal HCV screening would be both feasible and acceptable to most pregnant women attending antenatal clinics. The awareness of HBV and HIV (75%) was better than HCV (55%). Following written information and discussion, 99% of pregnant women were in favor of antenatal screening. Also, 96% found that the information they received about HCV was useful and felt that the delivery of this was carried out in a manner acceptable to the antenatal mothers. Antenatal screening would identify HCV-positive mothers and allow follow-up of their infants so that any infected mothers and infants could be offered effective curative therapy and prevent the progression of liver disease. The inclusion of HCV antenatal screening would complete the BBV profile and enhance the WHO target to eliminate HCV in the UK.

## Figures and Tables

**Table 1:** Patients' responses to questions addressing their understanding of viral screening and their views on routine screening for HCV in pregnancy (*n* = 420).

Question set 1						
	Yes		No		Not stated	

Did you know about hepatitis B, human immunodeficiency virus, or other infections tested during pregnancy?	318 (76%)		102 (24%)		0	
Have you ever heard of hepatitis C before this study?	232 (55%)		188 (45%)		0	
Did you agree to be tested for hepatitis C?	413 (98.4%)		6 (1.4%)		1 (0.2%)	
Would you be happy to have a hepatitis C test in a future pregnancy?	404 (96%)		13 (3%)		3 (0.7%)	
Should hepatitis C testing be available to all pregnant women?	415 (98.8%)		3 (0.7%)		2 (0.4%)	

Question set 2						
	Strongly disagree	Disagree	Neutral	Agree	Strongly agree	Not stated

Did you think the hepatitis C information that was discussed with you was useful?	0	4 (0.9%)	28 (6.7%)	104 (24.8%)	281 (66.9%)	3 (0.7%)
Did you think the hepatitis C information was discussed in a manner that was acceptable to you?	1 (0.2%)	1 (0.2%)	12 (2.9%)	86 (20.5%)	317 (75.4%)	3 (0.7%)

## Data Availability

Research data are not shared due to privacy and ethical restrictions.
